# A simple and efficient method for isolating small RNAs from different plant species

**DOI:** 10.1186/1746-4811-7-4

**Published:** 2011-02-24

**Authors:** Flor de Fátima Rosas-Cárdenas, Noé Durán-Figueroa, Jean-Philippe Vielle-Calzada, Andrés Cruz-Hernández, Nayelli Marsch-Martínez, Stefan de Folter

**Affiliations:** 1 Laboratorio Nacional de Genómica para la Biodiversidad (LANGEBIO), CINVESTAV-IPN, Km. 9.6 Libramiento Norte, Carretera Irapuato-León, CP 36821 Irapuato, Guanajuato, México; 2 Facultad de Ciencias Naturales-Biología, Universidad Autónoma de Querétaro, CP 76360 Juriquilla, Querétaro, México

## Abstract

**Background:**

Small RNAs emerged over the last decade as key regulators in diverse biological processes in eukaryotic organisms. To identify and study small RNAs, good and efficient protocols are necessary to isolate them, which sometimes may be challenging due to the composition of specific tissues of certain plant species. Here we describe a simple and efficient method to isolate small RNAs from different plant species.

**Results:**

We developed a simple and efficient method to isolate small RNAs from different plant species by first comparing different total RNA extraction protocols, followed by streamlining the best one, finally resulting in a small RNA extraction method that has no need of first total RNA extraction and is not based on the commercially available TRIzol^® ^Reagent or columns. This small RNA extraction method not only works well for plant tissues with high polysaccharide content, like cactus, agave, banana, and tomato, but also for plant species like Arabidopsis or tobacco. Furthermore, the obtained small RNA samples were successfully used in northern blot assays.

**Conclusion:**

Here we provide a simple and efficient method to isolate small RNAs from different plant species, such as cactus, agave, banana, tomato, Arabidopsis, and tobacco, and the small RNAs from this simplified and low cost method is suitable for downstream handling like northern blot assays.

## Introduction

Over the last decade small RNAs (sRNAs) have arisen as key regulators of diverse biological processes in eukaryotic organisms, including for instance development or stress responses, among others (reviewed in: [1-4]). sRNAs are around 20-30 nucleotide (nt) long, and guide regulatory processes at the RNA or DNA level.

The presence of endogenous sRNAs is now reported for many model plants and various non-model species (e.g. [5,6]), and elucidating the functions for many of these sRNAs will be a challenge in the near future. Two major classes of sRNAs are microRNAs (miRNAs; 21 and 24 nt long) and small-interfering RNAs (siRNAs; 18-24 nt long), with the latter being the most abundant, though, functionally less understood.

Various protocols are available for sRNA isolation from plants (e.g. [6-14]), though most of them are used for model plant species. Normally, these protocols start with total RNA isolation, followed by the isolation or separation of the low molecular weight RNA species (LMW RNA), containing the sRNAs. The most commonly used protocol is based on the extraction of total RNA using TRIzol^® ^Reagent [15,16] followed by precipitation of LMW RNAs using polyethylene glycol, and finally resulting in RNA species less than 300 nt long. Another protocol for total RNA isolation from tissues with higher contents of polysaccharides is the cetyltrimethylamonium bromide (CTAB) method [7,8]. These are useful protocols, but sometimes it is possible that these protocols do not work well for other plants species or specific tissues, or become quite labor-intensive due to difficult handling and the need of extra precipitation steps.

This motivated us to investigate whether it would be possible to find a generic protocol to isolate sRNAs, which would also work for plant tissues with a high polysaccharide content. In this report, a sRNA isolation method is presented that works efficiently for different plant species like cactus, banana and tomato fruits, and agave leaves, but also for Arabidopsis and tobacco with less polysaccharide content. The method presented here is not based on the use of the TRIzol^® ^Reagent or commercial columns and omits the total RNA isolation step and, therefore, becomes a simpler and cheaper sRNA isolation method for plants.

## Materials and methods

### Plant material

Prickly pear (*Opuntia robusta*) cactus pads and floral buds were collected at INIFAP (Mexican National Institute of Forestry, Agriculture, and Livestock Research) Campo Experimental Norte de Guanajuato, in San Luis de la Paz, Gto., Mexico. Agave leaves were collected at the campus of CINVESTAV, Irapuato, Gto., Mexico. *Arabidopsis thaliana *(ecotype Ws-3) and *Nicotiana tabacum *plants were grown under conventional long day growth conditions (22°C, 16 hours of light). Banana and tomato fruits were purchased at the local market. The samples were sliced, ground to a fine powder in a mortar with liquid nitrogen and stored at -80°C until further use.

### Buffers and solutions

#### • LiCl extraction buffer

100 mM Tris-HCl, pH 9.0

1% SDS

100 mM LiCl

10 mM EDTA

#### • TBE buffer (1x)

0.9 M Tris-HCl, pH 8.0

0.9 M Boric Acid

2 mM EDTA, pH 8.0

#### • Loading buffer

98% formamide

10 mM EDTA, pH 8.0

1 mg/ml xylene cyanol

1 mg/ml bromophenol blue

#### • Polyacrylamide stock solution

12.5% polyacrylamide (Acrylamide:bisacrylamide 19:1; Biorad)

0.5× TBE buffer, pH 8.0

7 M Urea

#### • Denaturing polyacrylamide gel (for one gel)

5 ml of polyacrylamide stock

25 μl of 20% APS (Ammonium persulfate)

5 μl TEMED (N, N, N', N'-Tetramethylethylenediamine)

#### • Staining solution

0.001% SYBR Gold (Invitrogen)

0.5× TBE buffer, pH 8.0

#### Other solutions

• 3 M sodium acetate, pH 5.2

• absolute ethanol

• phenol, pH 8.0

• chloroform-isoamyl alcohol (24:1; v/v)

• phenol-chloroform-isoamyl alcohol (25:24:1; v/v/v)

• 5 M NaCl solution

• 40% polyethylene glycol 8000 solution (PEG 8000)

• DEPC treated (0.05%) water

### For northern blot analysis

#### • EDC fixation solution (24 ml)

245 μl of 12.5 M methylimidazole, pH 8.0

0.5 g 1-ethyl-3-(3-dimethylaminopropyl) carbodiimide (EDC)

#### • Hybridization solution (100 ml)

10 g dextran sulphate

5 ml of 20% SDS

20 ml of 5 M NaCl

5 ml of 1 M Tris-HCl, pH 7.5

#### • Wash solution

2× SSC

0.1% SDS

## Protocol

### Small RNA extraction

1. Place 0.1 g of pulverized frozen tissue in a 1.5 ml microcentrifuge tube and add 500 μl of LiCl extraction buffer and 500 μl of phenol pH 8.0.

2. Shake or mix well using a vortex for 1 min. Place each sample on ice until all samples are ready.

3. Incubate tubes for 5 min at 60°C.

4. Centrifuge for 10 min in a microcentrifuge at max speed at 4°C.

5. Transfer the upper phase to a new microcentrifuge tube and add 600 μl of chloroform-isoamyl alcohol (24:1; v/v).

6. Centrifuge 10 min at max speed at 4°C.

7. Transfer the upper phase to a new microcentrifuge tube and incubate for 15 min at 65°C.

8. Add 50 μl of 5 M NaCl and 63 μl of 40% polyethylene glycol 8000 (w/v) and mix using a vortex for 1 min, followed by incubation on ice for at least 30 min.

9. Centrifuge for 10 min at max speed at 4°C (Note: the supernatant contains LMW RNA and the pellet consists of HMW RNA and DNA).

10. Transfer supernatant to a new microcentrifuge tube and add 500 μl of phenol-chloroform-isoamyl alcohol (25:24:1; v/v/v).

11. Centrifuge for 10 min at max speed at 4°C.

12. Transfer supernatant to a new microcentrifuge tube and precipitate LMW RNA by adding 50 μl of 3 M sodium acetate pH 5.2 and 1200 μl of absolute ethanol.

13. Incubate overnight at -20°C.

14. Centrifuge for 10 min at max speed at 4°C.

15. Discard supernatant and dry pellet. When dry, resuspend in 20 μl RNAse-free water.

16. Determine RNA purity and concentration by measuring their absorbance at 230, 260 and 280 nm, and calculate the A260/A280 and A260/A230 ratios.

### Small RNA analysis in polyacrylamide gel

1. Prepare a denaturing 12.5% polyacrylamide gel by mixing all components (see Materials and Methods; vertical electrophoresis gel system; work RNAse-free). Let the polyacrylamide polymerize for at least 30 min and then remove combs, gels may be stored at 4°C. Note: Polymerization time affects the quality of the run, and we have noted that gels prepared two days previous to their use showed improved band definition.

2. Pre-run the gel(s) in 0.5× TBE buffer (to remove ammonium persulfate residues) for 2 h at 90 V.

3. Prepare samples. For 2 μg LMW RNA, add 0.3 (v/v) loading buffer (adjust to the same volume in all samples with RNAse-free water). Incubate samples for 5 min at 65°C to denature RNA and immediately place on ice for at least for 1 min.

4. Before loading each sample in the gel, wash each gel slot with 0.5× TBE using a syringe.

5. Load the samples in the gel (fill empty slots with loading buffer) and run for around 2 h at 90 V in 0.5× TBE buffer (until bromophenol blue of the loading buffer reaches the end of the gel).

6. When the electrophoresis run is ready, take the gel out the chamber and stain for 30 min in 15 ml 0.5× TBE buffer with 0.001% SYBR Gold. Afterwards, rinse for 5 min with RNAse-free water.

7. Visualize the gel under UV light.

### Northern blot analysis

1. The gel may be used for northern blot analysis. In this report, the northern blot analysis was performed following the protocol by Pall and Hamilton (2008) [17] with modifications. Use a semidry trans-blot system (Biorad) to transfer the gel to a neutral nylon membrane (Hybond-NX, GE Healthcare) in 0.5× TBE buffer for 1 h at 10 V. Air dry the membrane at room temperature, add 12 ml of freshly made EDC fixation solution, incubate the membrane for 30 min at 60°C, and then rinse twice with RNAse-free water. Repeat this fixation step once more. Let the membrane dry and store at -20°C till further use.

2. Pre-hybridize with 15 ml hybridization solution (containing denatured salm sperm DNA) for 1.5 h at 60°C, followed by replacing the hybridization solution and adding the labelled probe of interest, and incubate for 24 h at 60°C. In this report, two probes were used (5'-AGGGGCCATGCTAATCTTCTC-3' and 5'-AAGAGCTCCCTTCAATCCAAA-3'), each labelled with [γ-32P]ATP to detect the small nucleolar RNA U6 and miRNA159a, respectively.

3. Wash the membrane twice with wash solution (first for 4 min, and then a second time for 2 min) at room temperature, followed by exposure to a storage phosphor screen for ~48 h at room temperature.

## Comments

### Total RNA isolation

The isolation of total RNA is a common step prior to LMW RNA isolation. Three different total RNA isolation protocols were compared: TRIzol^® ^(Invitrogen, Carlsbad, CA), CTAB [7], and LiCl [18]. We performed the comparison using a difficult (regarding RNA isolation) plant species, namely the prickly pear cactus (*Opuntia robusta*), because there are already many examples of successful total RNA isolation for model plants like Arabidopsis.

**Figure 1:**
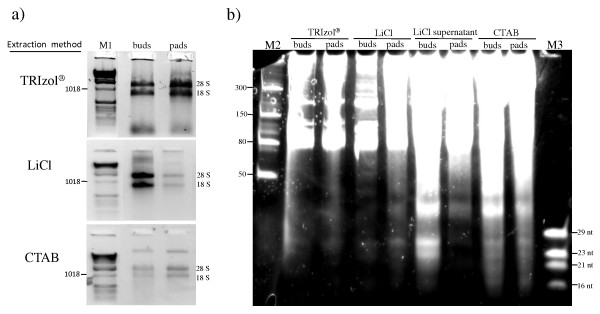
**Comparison of total RNA and low-molecular-weight (LMW) RNA samples isolated from cactus (*Opuntia robusta*) with different extraction methods**. a) Total RNA was extracted (in triplicate) with three different RNA extraction methods. 0.1 g of tissue was used for each sample and 1/10 volume of each sample was loaded on a 1% agarose gel (gel stained with ethidium bromide). b) LMW RNA extracted from total RNA samples obtained with different extraction methods, 1/10 volume (1-3 ug) of each sample was loaded on a 12.5% polyacrylamide gel (gel stained with SYBR gold). M1, 1 kb DNA ladder (Invitrogen); M2, Low Range ssRNA Ladder (NEB); and M3, oligonucleotide ladder.

Total RNA was isolated from cactus pads and floral buds as shown in Figure 1a. Differences in yield and quality were observed with respect to the tissue type. Total RNA was best isolated from cactus pads using TRIzol^® ^Reagent, while total RNA from floral buds was best isolated using the LiCl protocol. The 260/280 ratios were similar among the different methods (1.76-2.19), as presented in Table 1. However, 260/230 ratios showed large variations (0.21-2.03), being especially low when TRIzol^® ^Reagent was used. Possibly, guanidine thyocianate or other contaminants could be producing this effect. Electrophoretic analysis showed that the LiCl method produced the best RNA quality (Figure 1a). Moreover, this method was easier in comparison with the CTAB and TRIzol^® ^methods, as the mucilage, present in the cactus tissue, greatly difficulted handling and separation of the upper phases, requiring considerably long times for these steps.

**Table 1:** Concentration* of total RNA and LMW RNA samples obtained using different extraction methods

Method	Tissue	Average 260/280	Average 260/230	total RNA yield (μg)	260/280	260/230	LWM RNA yield (μg)
TRIzol^®^	buds	1.84	0.21	76.3	1.96	1.73	56.34
	pads	1.83	0.25	50.28	1.96	1.59	38.4
							
LiCl	buds	2.19	2.03	34.18	1.85	2.25	16.33
	pads	1.76	1.72	4.20	1.85	2.24	1.57
							
LiCl supernant	buds	---	---	---	2.03	1.94	29.88
	pads	---	---	---	1.87	1.61	24.42
							
CTAB	buds	2.19	2.01	24.65	2.12	2.22	16.81
	pads	1.86	1.29	17.60	2.05	2.15	10.26

*Yields and spectrophotometric A260/A230 and A260/A280 ratios of total RNA and LMW RNA samples extracted from cactus floral buds and pads with different extraction methods. 1 g of tissue was used for each total RNA extraction (in triplicate) and quantified in a Nanodrop spectrophotometer, followed by LMW RNA extraction (from 3 pooled total RNA samples).

### Small RNA enrichment

sRNA enrichment consists in the separation of RNA in high and low molecular weight species (which can be used for further analysis in gels, library construction, or direct sequencing). Besides comparing the different protocols of total RNA extraction for the more difficult cactus plant (Figure 1a), it was also analyzed whether the total RNA isolated using the different extraction methods finally results in a difference in LMW RNA enrichment. For this, total RNA was isolated using the three different extraction methods (TRIzol^®^, CTAB, and LiCl) and enriched for LMW RNA (step 8-15 of *small RNA extraction *protocol). A positive relationship between the quality and yield of LMW RNA and total RNA obtained by the different methods was observed (Figure 1b and Table 1). The supernatant obtained in the LiCl method was also analyzed, since it is assumed that sRNAs do not co-precipitate well with the HMW RNAs [11]. Indeed, this fraction contained high amounts of LMW RNAs, especially when isolated from floral buds (as shown in Figure 1b). Both the tissue type and the isolation method contributed to the yield. From the isolation method comparison, it was concluded that LMW RNA from the LiCl supernatants, and total RNA obtained with the LiCl method showed higher yields and quality.

**Figure 2:**
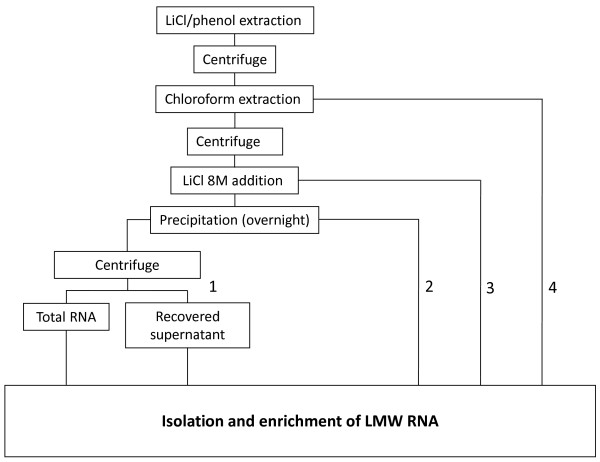
**Overview of the LiCl extraction method modifications**. 1) LMW RNA enrichment from the supernatants obtained after precipitation of total RNA, 2) LiCl overnight precipitation, followed by LMW RNA enrichment, 3) LiCl addition, immediately followed by enrichment of LMW RNA, and 4) Enrichment of LMW RNA directly after the chloroform extraction, omitting the LiCl precipitation step.

**Figure 3:**
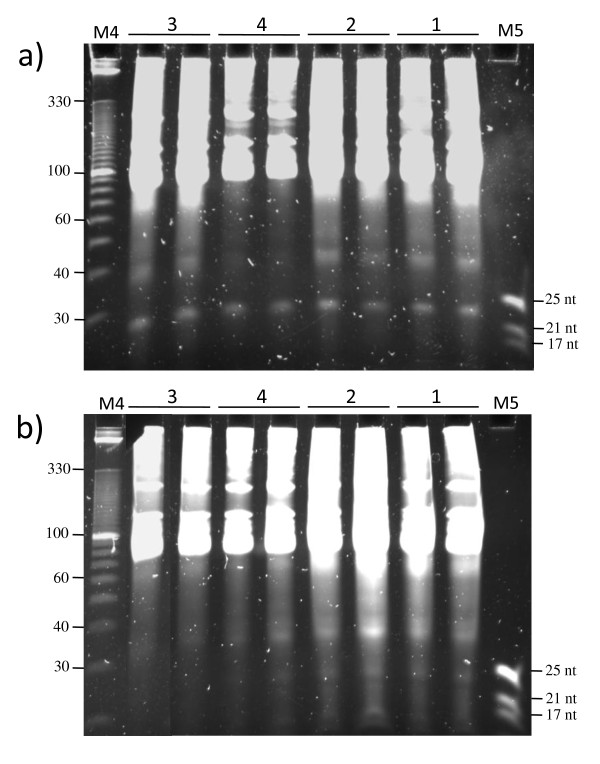
**Analysis of small RNAs isolated from cactus and Arabidopsis**. a) Gel electrophoresis of LMW RNA from cactus floral buds obtained with different modifications of the LiCl method, b) Gel electrophoresis of LMW RNA from Arabidopsis leaves obtained with various modifications of the LiCl method. Modification steps are indicated above each gel (a and b), namely 1) LMW RNA enrichment from the supernatants obtained after precipitation of total RNA, 2) LiCl precipitating, followed by LMW RNA enrichment, 3) Addition of LiCl, directly followed by LMW RNA enrichment, and 4) Enrichment of LMW RNA directly after the chloroform extraction without addition of LiCl. 0.1 g of tissue was used for each sample extraction (in triplicate). 2 μg of each sample was loaded on a 12.5% polyacrylamide gel (gel stained with SYBR gold). M4, 10 bp DNA Ladder (Invitrogen); M5, microRNA Marker (NEB).

As sRNAs were obtained both from the supernatant and the total RNA pellet (Figure 1b), it was tested whether the extraction could be streamlined avoiding the total RNA separation step. Different strategies were tested to optimize the extraction protocol in a way that sRNAs could be directly obtained by omitting the LiCl total RNA precipitation step. For this, the original LiCl protocol [18] was modified in four different ways by consequentially removing steps (Figure 2). As a control, the modifications were also tested with the model plant Arabidopsis. In the first modification, the enrichment step was performed using the supernatant directly after total RNA precipitation (without using the pellet containing HMW RNA), which produced positive results (Figure 3a and 3b). In the second modification, LMW enrichment was performed directly after the overnight LiCl precipitation. In the third modification, enrichment was done directly after the addition of LiCl. Finally, and since good LMW RNA yields were still obtained by removing the various steps, the complete LiCl precipitation step was omitted in the fourth modification. LMW RNA enrichment was directly performed using the upper phase after the chloroform extraction (modification 4 in Figure 2), which, strikingly, worked very well for both plant species (Figure 3a and 3b).

**Figure 4:**
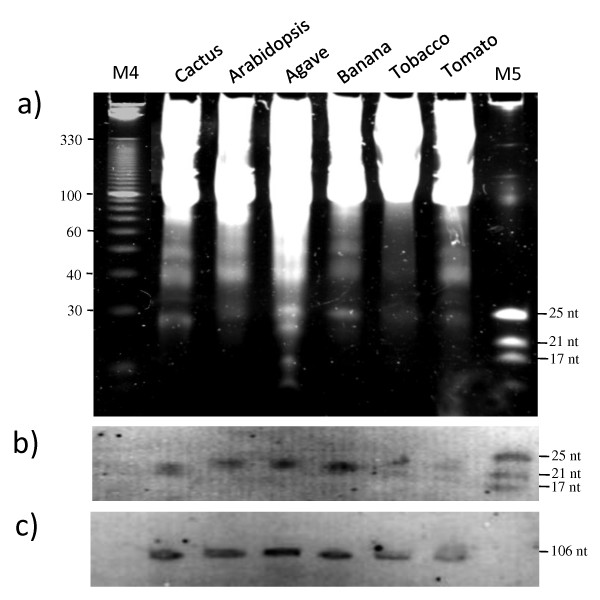
**Visualization and northern blot assays of small RNAs isolated from different plant species**. a) Gel electrophoresis of LMW RNA from cactus (floral buds), Arabidopsis (leaves), agave (leaf), banana (skin of the fruit), tobacco (leaf), and tomato (fruit) isolated (triplicate) with the optimized LiCl method (omitting the LiCl precipitation step; Figure 2-modification 4), b) Northern blot assay with de miRNA159a, and c) with the small nucleolar RNA U6 probe (same samples as in the gel presented in a)). 0.1 g of tissue was used for each sample extraction (in triplicate). 2 μg of each sample was loaded on a 12.5% polyacrylamide gel. M4, 10 bp DNA Ladder (Invitrogen); M5, microRNA Marker (NEB).

The extractions were performed in triplicate and the LMW RNA extracts were electrophoresed using denaturing polyacrylamide gels (Figure 3). The presence of sRNAs obtained with the different strategies was observed. The final strategy (where the enrichment step was performed right after the chloroform extraction) showed a less intense band corresponding to 40 nt RNAs and produced a cleaner extract compared to the other methods (Figure 3 and Table 2). Furthermore, spectrophotometric analysis showed that all the 260/280 ratios were high when using the different methods to extract LMW RNA from cactus tissue, indicating a good purity of the samples (Table 2). However, the 260/230 ratios were very low in all cases, both for cactus as for Arabidopsis. Nevertheless, LMW RNA analyzed in polyacrylamide gels showed well defined 5.8S, 5S, and tRNA bands, which suggests a good recovery (Figure 3). Moreover, a well defined 24 nt band (sRNAs) could be directly observed in the polyacrylamide gel. This band was observed and well defined when using the different methods. Interestingly, the sRNAs isolated using modification 4 (where the LiCl precipitation step was omitted) showed less background in the gel and a higher concentration of LMW RNA, showing that this strategy is efficient for LMW RNA isolation from plant species with high polysaccharide content, leading to increased yield in a reduced number of steps using conventional lab chemicals. This method does not only allow efficient LMW RNA recovery from cactus or Arabidopsis, but it also works very well for other plant species, such as agave, banana, tomato, and tobacco (Figure 4a). Furthermore, when necessary, HMW RNA may still be recovered from the pellet after the polyethylene glycol precipitation (step 9 of the *Small RNA extraction *protocol; data not shown).

**Table 2:** Concentrations* of LWM RNA samples obtained using the LiCl method with different modifications

Tissue	Modification	ng/μl	Average 260/280	Average 260/230	Yield (μg)
Cactus	1) LMW RNA enrichment from the supernatants obtained after precipitation of total RNA	382.30	1.82	1.81	7.65
	2) LiCl precipitating (overnight), followed by LMW RNA enrichment	527.75	1.81	1.90	10.56
	3) Addition of LiCl, directly followed by LMW RNA enrichment	291.42	1.66	1.35	5.83
	4) Enrichment of LMW RNA directly after the chloroform extraction without addition of LiCl	1008.42	2.02	1.04	20.17
					
Arabidopsis	1) LMW RNA enrichment from the supernatants obtained after precipitation of total RNA	582.52	1.68	1.23	11.65
	2) LiCl precipitating (overnight), followed by LMW RNA enrichment	333.68	1.66	1.21	6.67
	3) Addition of LiCl, directly followed by LMW RNA enrichment	609.38	1.53	1.13	12.19
	4) Enrichment of LMW RNA directly after the chloroform extraction without addition of LiCl	1418.77	1.53	0.96	28.38

*Yields and spectrophotometric A260/A230 and A260/A280 ratios of LMW RNA isolated with LiCl extraction method modifications from Prickly pear cactus and Arabidopsis. 0.1 g of tissue was used for the extraction and quantified using a Nanodrop spectrophotometer. LMW RNA was extracted two times in triplicate (6 independent samples).

### Northern blot analysis

As described above, positive results were obtained for the isolation of LMW RNAs using the fourth modification and well defined sRNA bands could be visualized in a gel for 6 different plant species (Figure 4a). To test whether the LMW RNA observed in the gel represented intact molecules and not degradation products, a northern blot assay was performed. Figure 4b and 4c shows that indeed, LMW RNA from cactus and Arabidopsis isolated with the shortest protocol version can be successfully detected in northern blot hybridization experiments using the miRNA159a probe, detecting 21 nt sRNA molecules, as well as using the small nucleolar RNA U6 probe, which detects 106 nt LMW RNA species (Figure 4b and 4c, respectively).

## Conclusion

Here we provide a simple and effective method suitable for sRNA extraction from polysaccharide-rich material such as cactus, agave, banana, and tomato tissues, which also works well for less complex plant tissues form e.g., Arabidopsis and tobacco. This modified extraction method gives good yield and quality of LMW RNA species. Moreover, the LMW RNA obtained from the different plant species was successfully used for northern blot assays. Well defined bands were detected when using the miRNA159a and the small nucleolar RNA U6 probes. Therefore, the sRNA molecules that can be obtained with this low-cost short method are suitable for downstream assays like northern blot hybridization, and most likely also for cloning and sequencing of sRNAs.

## Competing interests

The authors declare that they have no competing interests.

## Authors' contributions

FFRC did the major experimental work. NDF and JPVC contributed to the northern blot assays and with technical advice. FFRC, ACH, NMM, and SDF conceived the project and designed the experiments. FFRC, NMM, and SDF drafted the manuscript. All authors read and approved the final manuscript.
